# The association between intelligence and lifespan is mostly genetic

**DOI:** 10.1093/ije/dyv112

**Published:** 2015-07-26

**Authors:** Rosalind Arden, Michelle Luciano, Ian J Deary, Chandra A Reynolds, Nancy L Pedersen, Brenda L Plassman, Matt McGue, Kaare Christensen, Peter M Visscher

**Affiliations:** ^1^ Centre for Philosophy of Natural & Social Science, London School of Economics, London, UK,; ^2^ Genetic Epidemiology, Queensland Institute of Medical Research, Brisbane, QLD, Australia,; ^3^ Centre for Cognitive Ageing and Cognitive Epidemiology, University of Edinburgh, Edinburgh, UK,; ^4^ Department of Psychology, University of California, Riverside, CA, USA,; ^5^ Department of Medical Epidemiology and Biostatistics, Karolinska Institutet, Stockholm, Sweden,; ^6^ Department of Psychiatry and Behavioral Sciences, Duke University Medical Center, Durham, NC, USA,; ^7^ Department of Psychology, University of Minnesota, Minneapolis, MN, USA,; ^8^ Danish Ageing Research Center, University of Southern Denmark, Odense, Denmark,; ^9^ Danish Twin Registry, University of Southern Denmark, Odense, Denmark and; ^10^ Queensland Brain Institute, University of Queensland Diamantina Institute, Translational Research Institute, Woolloongabba, QLD, Australia

## Abstract

**Background:** Several studies in the new field of cognitive epidemiology have shown that higher intelligence predicts longer lifespan. This positive correlation might arise from socioeconomic status influencing both intelligence and health; intelligence leading to better health behaviours; and/or some shared genetic factors influencing both intelligence and health. Distinguishing among these hypotheses is crucial for medicine and public health, but can only be accomplished by studying a genetically informative sample.

**Methods:** We analysed data from three genetically informative samples containing information on intelligence and mortality: Sample 1, 377 pairs of male veterans from the NAS-NRC US World War II Twin Registry; Sample 2, 246 pairs of twins from the Swedish Twin Registry; and Sample 3, 784 pairs of twins from the Danish Twin Registry. The age at which intelligence was measured differed between the samples. We used three methods of genetic analysis to examine the relationship between intelligence and lifespan: we calculated the proportion of the more intelligent twins who outlived their co-twin; we regressed within-twin-pair lifespan differences on within-twin-pair intelligence differences; and we used the resulting regression coefficients to model the additive genetic covariance. We conducted a meta-analysis of the regression coefficients across the three samples.

**Results:** The combined (and all three individual samples) showed a small positive phenotypic correlation between intelligence and lifespan. In the combined sample observed *r* = .12 (95% confidence interval .06 to .18). The additive genetic covariance model supported a genetic relationship between intelligence and lifespan. In the combined sample the genetic contribution to the covariance was 95%; in the US study, 84%; in the Swedish study, 86%, and in the Danish study, 85%.

**Conclusions:** The finding of common genetic effects between lifespan and intelligence has important implications for public health, and for those interested in the genetics of intelligence, lifespan or inequalities in health outcomes including lifespan.

Key Messages

- It has been reported that brighter people live longer; we asked ‘why?'.
- We found, using data from three studies, that the small association between being brighter and living longer was mostly genetic in origin.
- This is a key finding in cognitive epidemiology; it is a further indication that intelligence is not just ‘school-smarts'.

## Introduction

In 1991, a study of the health of British civil servants showed that even under nationalized health care, and among the employed, people at the bottom of the job hierarchy have an annual 3-fold higher risk of all-cause mortality compared with those at the top. ^1^ A similar positive association with mortality has been reported for measured intelligence. A population study of people born in Scotland in 1921 showed that intelligence measured at age 11 predicted survival to age 76. ^2^ The same intelligence-lifespan association has now been replicated in other studies. ^3^ These findings have given rise to a large literature on mortality inequalities, and the associations among intelligence, socioeconomic status, health behaviours and lifespan. ^4^^,^^5^ What causes the relationship between intelligence and lifespan? Factors such as rearing environment, family income, schooling, lifestyle choices, or constraints such as diet, exercise, accidents, illnesses, ^2^^,^^5^ may each play a role.

There are several mutually compatible explanations for the covariance between intelligence and lifespan. Higher intelligence could cause longer lifespan through mediators such as higher income, safer employment or better health choices. Although early rearing is important, ^6^ there is good evidence that, within the normal range of families, rearing environments do not explain the variation among people in intelligence measured after adolescence. ^4^^,^^7^^,^^8^ So the shared environment—what makes people within a family more similar to one another and contributes to between-family differences—is an unlikely cause of the covariance between the two traits, intelligence and lifespan. Perhaps more likely is that shared genetic factors may act on both traits. Analytical designs that test between monozygotic (MZ) twins that share all their segregating genes and dizygotic (DZ) twins that share around half their genes, are useful to probe the covariance between intelligence and lifespan. ^7^ If shared genetic factors influence both traits, ^8^^,^^9^ we should see a positive association between intelligence and lifespan differences within DZ twin pairs. Differences within DZ twin pairs are due to genes and individual-specific non-genetic factors that may influence both intelligence and lifespan. But we would not expect to see an association between intelligence and lifespan differences within MZ twin pairs (who like DZ twins are also matched on rearing environments) but whose differences are due only to individual-specific non-genetic factors. These are, of course, not the only possible set of relationships. Evidence of gene-environment correlations ^10^^,^^11^ prepares us to expect that genes contributing to intelligence may be associated with environments that promote health. Genes that contribute to good cognitive abilities may also influence health-promoting decision making.

We turned to the most revealing datasets we could identify to test the weight of the available evidence—these are twin samples where both intelligence and mortality have been recorded and where at least one twin within a pair had died. Our objective was to discover whether there is any evidence that genetic factors directly influence the covariance of intelligence and mortality. In our results and discussion we refer to lifespan rather than ‘mortality’ to avoid repeated negatives (as in ‘higher intelligence is negatively correlated with mortality’).

## Methods

### Samples

#### Study 1: US Military veteran sample

The NAS-NRC Twin Registry of WWII male veterans in the USA was initially compiled by matching records of multiple births, from 1917 to 1927 in 42 states, with military service records. The current sample included 377 (201 MZ) twin pairs whose military entrance examination scores were available and at least one member of the twin pair was deceased. At the time of enlistment in the military, each man (aged between 18 and 25 in the 1940s) took an entrance test [either the Army General Classification Test (AGCT) or the General Classification Test (GCT)]. Our sample comprised 579 men who had died by 2009, as well as 175 living men. Among 202 pairs, both twins had died (mean age of death 72, range 43–92 rounded). In 175 pairs, one twin had died (mean age of death 77, SD 5.4, minimum 64, maximum 88). Where one member was alive, we imputed his life expectancy based on a life table. ^12^ If both twins were living, the actuarial estimated life expectancy would have been the same for both twins, ruling out any life expectancy differences.

#### Study 2: Swedish twin sample

We examined data from 790 men and women from the Swedish Adoption/Twin Study of Aging (SATSA) for whom general intelligence test scores were available based on completion of multiple cognitive tests. Mortality data were collected in May 2014 from a national death registry. Within this sample of same-sex twins, we selected only the twin pairs in which at least one person had died, yielding 246 pairs (111 male pairs, 135 female pairs, 100 MZ), all born between 1900 and 1939. Among 164 pairs, both twins had died (mean age of death 84, range 59–104 rounded). In 82 pairs, one twin had died (mean age of death 77, minimum 57, maximum 105). For the surviving twins, we imputed a date of death. Statistics Sweden provided current life tables from which we could calculate the actuarial life expectancy for each living twin. ^13^ The index of intelligence in our analyses was the first unrotated principal component extracted from the scores of 12 verbal and non-verbal tests of cognitive ability. The minimum recruitment age was 50; the cognitive tests were administered at a mean age of 66 years.

#### Study 3: Longitudinal Study of Aging in Danish Twins sample (LSADT)

This population-based sample is a subset from the oldest nationwide twin registry: the Danish Twin Registry. ^14^ The study comprises 784 male and female twin pairs (305 MZ pairs) born between 1920 and 1930. Twins entered the study having survived to at least age 70. Mortality was established in 2012. We included only complete pairs of twins, and those pairs where at least one member of the pair had died. In 451 pairs, both twins had died (mean age of death 85, range 72–104 rounded). In 333 pairs, one twin had died (mean age of death 81, SD 5.4, minimum 71, maximum 96). Where one twin was alive we imputed their life expectancy from a Danish life table, by sex and birth year. ^15^ Sixteen individuals (aged > 93 years) exceeded their actuarial life expectancy within the study, so their current age was retained. Intelligence was indexed by a composite score derived from the following five tests: Fluency, Digit forwards, Digit backwards, Immediate recall and Delayed recall. ^16^ Cognitive abilities were assessed at a mean age of 76.

### Sex differences

In all three samples, the available data included only same-sex twins. During data preparation the main effect of sex was tested in an ANOVA on the absolute life expectancy differences in the Swedish and Danish samples.

### Analysis

We conducted the same analyses on the combined samples (using z-scores), and on all three samples separately (available as Supplementary data at *IJE* online). In the first analysis we asked: ‘Does the more intelligent twin in each pair live longer than their co-twin more often than expected?’ Within each pair we scored the brighter twin 1 and their co-twin 0. Next, within each pair we scored each twin ‘1’ if they lived longer than their co-twin, whom we scored ‘0’. Then, using a cross tabulated contingency table we examined the Pearson chi square statistic. This statistic quantifies the extent to which the concordance of the two dichotomous variables (lived longer / was brighter) differs from the expected value under the assumption of statistical independence. We re-ran the same contingency table procedure after stratifying the sample by zygosity (analysing the MZ twins in one group and the DZ twins in the second group). If aspects of the ‘unique environment’ mediated the association between intelligence and lifespan, we would expect the phenotypically brighter twin, within an MZ pair, to live longer on average. On the other hand, if the intelligence-lifespan association is driven by genes, we would expect no difference in longevity stratified by the intelligence of MZ co-twins who share the same genes. However, common genetic factors that influence both intelligence and lifespan would produce an over-representation of longer-living co-twins for the phenotypically brighter twin within DZ twin pairs, who share on average only 50% of genes.

We then turned to linear regressions. We calculated two standardized within-pair difference scores: the age-at-death difference and the intelligence difference score. We regressed the age-at-death difference score (as the outcome variable) on the intelligence difference score (as the predictor variable). The reasoning is as follows. First, if intelligence and lifespan co-vary for genetic reasons, we would expect that the lifespan difference within a pair of dizygotic twins would increase as a function of the within-pair intelligence difference. Second, MZ twins are genetically identical; if we find a significant regression within the MZ twins, we may infer that non-shared environmental effects contribute to the association. We expect a significant regression between the two difference scores, for genetic reasons, only among the DZ twins. So, we examined the linear regressions separately for the genetically identical MZ twins, and for the DZ twins who share half their segregating genes on average. We predicted a significant positive correlation between intelligence-difference and lifespan-difference scores within DZ twins (but not in MZ twins), exposing a genetic relationship between intelligence and lifespan.

In the third and last analysis, we tested an additive genetic (A) and unique environment (E) model to test for genetic influence on both intelligence and lifespan. The model specification is available as Supplementary data at *IJE* online. The AE model assumes that differences within twin families are caused by additive genetic and individual-specific, non-genetic factors. We implemented this model because of the repeated finding that the impact of shared environmental factors on intelligence declines steadily, even within childhood, ^17–19^ and is rarely discernible from adolescence onward. ^20–22^ The model depends upon the standardized coefficient from the linear regressions described above, together with their standard errors (SE), as well as the variances and covariance of the two traits.

In this model, the heritabilities of adult intelligence and longevity are derived empirically. To examine the average MZ/DZ difference across the three samples, we conducted a random-effects meta-analysis run in MCMCglmm using R ^23^ across the three regression coefficients. This analysis weights the study by the standard error of the regression coefficients and fits an effect for study heterogeneity.

Tests for the impact of outliers (more than three standard deviations from the mean) were conducted on the data, and on the difference scores in the combined sample. Since there was no substantive change to the results, we retained all the data to reduce the standard errors.

## Results

### Sex differences in life expectancy difference scores

Although there was a trend towards greater mean life expectancy difference scores among women, it was not significant: Swedish sample, F(1, 244) = 0.829, *P* = 0.363; Danish sample F(1, 782) = 3.112, *P* = 0.078.

### Combined samples and meta-analysis

When MZ and DZ twins were combined, we found that the brighter twin lived significantly longer than his/her co-twin in the pooled sample; this was also found in the Swedish and Danish samples. This is shown in the contingency table ( Table 1 ). Raw counts are given in Supplementary Data (available as Supplementary data at *IJE* online).

**Table 1.** Contingency table results (χ ^2^ test for the hypothesis that brighter twins live longer, shown separately for zygosity, and within all samples

	*N* pairs	Pearson $\chi_{1}^{2}$	*P* -value (exact 2-sided)
Combined samples			
MZ & DZ	1382	25.047	<0.001
MZ	614	2.170	0.141
DZ	768	29.308	<0.001
US military sample			
MZ & DZ	342	0.000	0.995
MZ	201	0.107	0.743
DZ	141	0.171	0.679
Swedish SATSA sample			
MZ & DZ	246	4.300	0.038
MZ	100	2.597	0.158
DZ	146	1.783	0.190
Danish LSADT sample			
MZ & DZ	782	31.184	<0.001
MZ	305	3.189	0.074
DZ	477	32.720	<0.001

The within-pair effect was non-significant in the MZ twins ( *P* = 0.36), but significant in the DZ twins ( *P* < 0.001). The heritability of the cognitive composite (intelligence) estimated from the combined sample was 0.52. The combined sample heritability of life expectancy was 0.28; this is closer to reports in the literature ^24^ than our individual samples (available as Supplementary data at *IJE* online).

**Figure 1.**
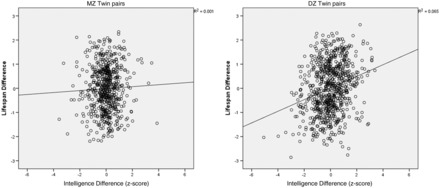
MZ (on left) and DZ (on right) twins. Regression of within-pair lifespan difference z-score on within-pair intelligence difference z-score (each datum = one within-pair difference score) in combined samples.

The within-twin-pair analysis of the life expectancy difference regressed on the intelligence difference was β = 0.04 (SE 0.05), *P* = 0.36 for the MZ twins. Among the DZ twins it was β = 0.25 (SE 0.04), *P* < 0.001. Across the whole sample, the regression gave β = 0.18, (SE 0.03), *P* < 0.001. Figure 1 shows a scatterplot with a best-fit regression line that shows the slope and direction of the within-pair intelligence-life expectancy relationship.

The regression analysis shows that twin pairs of both zygosities show the tendency of the brighter twin to live longer. However, this trend is significantly higher ( *P* < 0.001) among the DZ twins, supporting genetic mediation of the two traits (exact *P* values for the regressions and the MZ/DZ difference in slopes for individual samples are provided in Supplementary data , available at *IJE* online). The random-effects meta-analysis of the MZ and DZ regression coefficients across the three samples also confirmed a significant average difference (-0.20, SD = 0.10, *P* = 0.04) between MZ and DZ pairs in the regression coefficient.

The phenotypic correlation between intelligence and life expectancy [estimated at 0.32 (SE 0.07) under an AE model, shown in Table 2 below] was mostly explained by genes (95%).The observed phenotypic correlation between intelligence and life expectancy was 0.12.

**Table 2.** Summary of AE model results in combined and independent samples

Sample/ *N* pairs	AE model			Genetic contribution to phenotypic *r* intelligence/lifespan
	h ^2^ intelligence	h ^2^ lifespan	Phenotypic *r* intelligence/lifespan	
Combined/1312	0.52	0.27	0.32	95.0%
US military/377	0.60	0.06	0.16	83.7%
Swedish/188	0.98	0.22	0.26	86.3%
Danish/784	0.20	0.28	0.35	85.3%

The genetic contribution to phenotypic *r* is given by the genetic covariance between intelligence and life expectancy scores divided by the phenotypic covariance (both covariances are standardized).

## Discussion

In the first quantitative genetic study to analyse the association between intelligence and lifespan, we found evidence (summarized in Table 2 ) that the covariance between lifespan and intelligence is strongly influenced by genetic factors. Finding three genetically-informative samples containing a measure indexing intelligence, and where lifespan was known, was very useful; however, there are important limitations concerning our samples, discussed below.

We conducted several tests; they did not all yield significant results, nor was every result consistent across the three samples. Recruitment age, cognitive testing age and cognitive tests varied among the samples; this matters for comparability. The Danish sample is the largest and a driver of our key results, yet the empirical heritability of cognitive ability was low in that sample, possibly a combination of: tests that are less *g* -loaded (less correlated with the common variance among tests) and less heritable; ascertainment (twins entering the study had already survived to age 70, which may increase their cognitive similarity regardless of zygosity), and sampling variability. An ideal sample (for our research question) would include twins whose cognitive ability was measured in youth and again at young adulthood, together with mortality data. By contrast, twins in the Scandinavian samples entered the study at a later age. This means that we did not capture all the variance in life expectancy differences that would occur in the general population. Among the US veterans, the within-pair contingency tables showed no influence of genes on the relationship between intelligence and life expectancy; yet the regression analyses did support such a genetic influence on lifespan, and the tests on the combined sample were consistent with the meta-analysis across the three samples.

Our results here concern same-sex pairs, which avoid within-pair sex effects. Since women's average life expectancy exceeds men's average life expectancy, larger studies with earlier recruitment might find a greater mean among women's life expectancy difference scores.

We note that the causes of the association between intelligence and lifespan may vary between ages (especially since the causes of deaths differ by age). Further, cognition measured in older age is a combination of trait level of intelligence and the amount of cognitive decline. Older age recruitment in the Scandinavian samples will have caused range restriction in life expectancy scores, which means that the true size of the phenotypic correlation between cognitive ability and life expectancy may be larger than reported here.

Any genetic factors that contribute to intelligence and lifespan may operate indirectly via good health choices or higher income which leads to better healthcare in some countries. We note that these behaviours (intelligence, income, lifestyle choices) are themselves associated through gene-environment correlations. Such genetic relationships between intelligence and health-promoting behaviours have been reported. ^25^ An alternative (and compatible) genetic explanation relies on genetic pleiotropy. In cognitive epidemiology, the question ‘what causes the link between intelligence and lifespan?’ is unsolved and crucial. It matters for the tautological reason that evidence-based policy depends on evidence. These findings matter because they present a novel way of exploring socially important questions in public health. So far as we know, an empirical test of a direct genetic link between intelligence and lifespan is new to this study.

We have shown in our AE model that the small covariance between intelligence and lifespan is almost entirely genetic. Others have shown a phenotypic correlation between intelligence and brain resilience to systematic insults, ^26^ and genetic correlations between intelligence and traits as diverse as: total brain volume; ^27^ openness; ^28^ conscientiousness; ^29^ agreeableness; ^30^ low hyperactivity; ^31^ reaction-time consistency or speed; ^32^ height; ^33^ and capacity in the elderly to walk, run and climb stairs. ^34^ Recent research suggests that there is a general factor that predicts rank on intelligence-type tasks not just in humans, but also across species. ^35^ Further research will reveal whether this general factor of intelligence is genetically associated with ecologically relevant attributes such as fertility, health and lifespan in species that do not have wealth gaps or lifestyle choices. If so, there may be an overarching genetic fitness factor, that explains positive correlations across many brain and body traits, which would be common across species.

### Conclusion

The Nordic countries are exemplars of wealth redistribution. Evidence from them is often used to support the claim that narrow wealth gaps promote health and life expectancy. ^36^ From a broad population-level perspective this may be true. Yet our results show that the relationship between lifespan and intelligence (which predicts wealth, even within advantaged families ^37^ ) is mostly genetic. We should be mindful that intelligence may mediate apparent associations between levels of education, income or occupation and morbidity and mortality. Genetically informative studies permit an individual differences perspective that can illuminate surprising connections among the aetiologies of these traits. Our results should be of interest to epidemiologists and molecular geneticists. If these results generalize, then alleles favouring intelligence may also favour lifespan even if the heritability of lifespan is low. This is because evolution gains traction from even minute advantages; what matters is the robustness of the association over generations, not the size of the advantage. Genetically informative data have a critical role to play in cognitive epidemiology and public health.

## Funding

This work was supported by the University of Edinburgh Centre for Cognitive Ageing and Cognitive Epidemiology, part of the cross council Lifelong Health and Wellbeing Initiative (grant numbers MR/K026992/1) to IJD. Funding from the Biotechnology and Biological Sciences Research Council and Medical Research Council is gratefully acknowledged to IJD. The Duke Twins Study of Memory in Aging in the NAS-NRC WWII male veterans was funded by the National Institutes of Health/National Institute of Aging RO1 (grant number AG08549) to BLP. The Swedish Adoption and Twin Study of Ageing is supported by the National Institute on Aging (grant numbers AG04563, AG10175), the MacArthur Foundation Research Network on Successful Aging, the Swedish Council for Social Research (grant number 97:0147:1B), and the Swedish Research Council to NP. PMV is supported by the National Health and Medical Research Council (NHMRC) of Australia. The content is solely the responsibility of the authors and does not necessarily represent the official views of the NHMRC. The Danish Twin Registry work was supported by grants from The National Program for Research Infrastructure 2007 (09-063256) from the Danish Agency for Science Technology and Innovation, the Velux Foundation and the US National Institute of Health (P01 AG08761).

## Supplementary Material

Supplementary Data
