# Supplementary material for: The association between intelligence and lifespan is mostly genetic
Source: Int J Epidemiol. 2015 Jul 26;45(1):178–85. doi: 10.1093/ije/dyv112 (PMC4795559; doi:10.1093/ije/dyv112)
Supplement: Supplementary Data [file dyv112_supplementary_data.zip › ije-2014-10-1250-File002.docx]

**Supplementary Materials: The association between intelligence and lifespan is mostly genetic.**

**Table of Contents**

2. Methods. Sample characterisation

3. Methods. Descriptive checks of the data

3. Results. Detailed results of three independent samples

7. Discussion. Data considerations

8. Tables

11. AE Model specification

13. Figure S1

14. Figure S2

15. Figure S3

16. References for the Supplementary Materials

**Detailed sample characterisation of the three independent studies**

**Study 1. US Military veteran sample**

The Registry originally consisted of 15,924 white male twin pairs. As part of the process of developing the Registry, information, including military entrance exam scores, was abstracted from military records and stored as part of the individuals Registry data file. However, prior to the completion of the military record abstraction, a fire destroyed the original military records of many of the veterans. The GCT and AGCT IQ-type tests were implemented to place candidates for training in suitable trades within the Armed Forces. The minimum entry requirement varies somewhat between historical periods (it is lowered during wartime when the demand for serving men is higher), but men with a very low IQ-type score do not enter the military so this sample is not represented at the very low end of the intelligence distribution. It is possible that men who join the military are more risk-taking than men in the general population - which may influence their life expectancy.

**Study 2. Swedish twin sample**

We added the sex-specific life-expectancy to the person’s last known age. For example, a man aged 75 years had 11.22 additional years designated as his lifespan. The twins whose life-expectancies were imputed ranged from 59-100 years old in 2014.

Our index of intelligence was derived from subtests including the Information subtest, Synonyms, Analogies, Block Design, Figure Logic, Card Rotation, Thurstone’s Memory, Digits Forward, Names and Faces Immediate and Delayed Recall, Symbol Digit, Figure Identification (for details of test administration see ^1^). The tests were administered to the twins when their mean age was 66.4 years (range 50-87 years, given a minimum recruitment age of 50). Twin pairs in this analysis were tested within the same wave of data collection (mode of one month, maximum of six months apart).

**Study 3. Danish twin sample**

Sample description is given in the manuscript.

**Descriptive Checks of the Data**

We examined the distribution of the key variables: intelligence and lifespan (in all three samples) to check for systematic differences in the data distributions between twin ‘1’ and twin ‘2’, between zygosities, or between twins who were deceased versus twins whose lifespans were imputed according to the life-tables. We also examined the distribution of within-pair difference scores in intelligence and lifespan. We found one unexplained pattern. Among the pairs of US and Danish twins where one twin was deceased and the other twin’s lifespan was imputed from data with a lifetable, the within-pair death-differences were not as normally distributed as in the Swedish sample. In the US sample, the frequency table showed that there were no values (death-differences) below 3.4 years, for the Danes 2.4% of values were less than 3.4 years, whereas, in the Swedish sample this was 11%. We do not have an explanation for this descriptive finding. If there is a reporting or imputation bias on age at death of the longest surviving twin in the US cohort then it is unlikely to bias results on the association between lifespan and intelligence differences, because the latter was from measurements taken much earlier, at the time of entrance in the military.

**Further details from Results of the three independent studies**

**Note:** Intelligence scores in the Swedish and Danish samples (where there was variance in age of testing) were adjusted for age prior to analysis; standardized scores from each sample were calculated so that the scale was consistent between samples.

**Study 1. US Military veteran sample**

The contingency table compared the number of cases in which the brighter twin lived longer more often than would be expected by chance, tested using a chi-square statistic. Table 1 (manuscript) shows no evidence that the higher-scoring twin lived longer (note that twin pairs whose intelligence or lifespan was equal were excluded from these analyses). The χ^2^ value of 342 pairs was zero. Among the 201 MZ pairs the χ^2^ value of .11 was non significant (p=.74). Among the 141 DZ pairs the χ^2^ value of .17 was also non significant (p=.68). The heritability of the cognitive composite (intelligence) estimated from this sample was .60. The sample heritability of the life expectancy measure using the imputed death age was .06. Figure S1 shows a scatterplot with a best fit regression line that shows the slope and direction of the within pair intelligence-life expectancy relationship. Among the MZ twins where we expected no relationship the slope is small. The slope is greater among the DZ twins. This is as expected if the two traits are mediated by a direct genetic influence.

The regression of the within-pair lifespan differences on within-pair intelligence differences gave, for the MZ twins, the standardized regression coefficient β=-0.15, (SE) .08, p=.03. This means that among the MZ twins, the lower scoring twin had a longer lifespan. Among the DZ twins the standardized regression coefficient was β=.14 (SE .08), p=.09. This shows that among the DZ twins, the higher scoring twin had a longer lifespan. We tested whether the MZ and DZ coefficients were different from each other at the conventional level of significance and they were (*p*=.01). When we entered the regression coefficients into the AE model (where the heritability of intelligence was estimated empirically from the sample as .60, and the heritability of life-expectancy at .06), the phenotypic correlation between intelligence and life-span was estimated at .16 of which the additive genetic contribution to the covariance between intelligence and lifespan was 83.7%. The phenotypic positive correlation between intelligence and life-expectancy in the observed data was *r*=.13.

**Results. Study 2. Swedish sample**

The cross-tabulated contingency table analysis (Table 1, manuscript) demonstrates that when all twin pairs are considered together regardless of zygosity, intelligence and lifespan are associated positively within each pair: the more intelligent twin lived longer than expected. Among the whole sample of 246 pairs the χ^2^ value was significant (p=.04). Among the 100 MZ pairs the χ^2^ value was non-significant (p=.11). Among the 146 DZ pairs the χ^2^ value was not significant (p=.18).

The heritability of the cognitive composite (intelligence) estimated from the full sample was .98. The sample heritability of the life expectancy measure was .22.

The regression analysis followed the pattern of the contingency table proportions tests. Figure S2 shows scatterplots of the within-pair lifespan difference scores regressed on within-pair intelligence difference scores for MZ and DZ twins. The regression of the within-pair lifespan differences on within-pair intelligence differences gave, for the MZ twins, the standardized regression coefficient β=.20, (SE .14), *p*=.05. For the DZ twins, the standardized regression coefficient was β=.22 (SE .07), *p*<.01. Within both zygosities, the brighter twin lived longer. We tested whether the MZ and DZ coefficients were different from each other at the conventional level of significance and they were not (*p*=.91), so we do not rule out sampling error.

The AE model is fitted based on parameters from the regression of life-expectancy difference scores on intelligence difference scores and the co-twin correlations for intelligence in MZ and DZ twins. These enable us to partition the covariance between intelligence and life expectancy into A and E components. The percentage of the phenotypic correlation between intelligence and life-expectancy r=.26 (SE .11) estimated under an AE model that is explained by genes is 86.25%. We note that the observed phenotypic correlation between intelligence and life-expectancy in the sample was r=.09. The reported SE are lower-bound SE; the approximations of the SE of the estimates of the correlations assume that cognitive parameters (variances, heritability) are known without error, which is not true.

**Results. Study 3. Danish sample**

The contingency table (Table 1 manuscript) showed that within a twin pair the brighter twin also tended to live longer. Across the whole sample, the brighter twin also lived longer to an extent that was significant (p<.001). This was non-significant among the 305 MZ twin pairs (p=.07), but was highly significant among the 477 DZ twin pairs (p<.001), that is, the frequency with which the brighter twin lived longer exceeded expectations under a model of independence.

The heritability of the cognitive composite (intelligence) estimated from the sample was .20. This seems low, but has been re-checked. Several factors may combine to explain this low estimate. We used falconer's formula to calculate the heritability (which does not provide a measure of the standard error), but we examined the standard error of the genetic covariance estimate in the Danish sample to that of others; it does not sit outside the expected range (Swedish sample SE= .11, US Military sample SE=.08, Danish sample SE=: .09, Combined sample SE= : .06). Tests in this study comprised measures that are somewhat less heritable than those used in the other samples. The sample includes twins who had survived till age 70 and may have been more similar with respect to their cognitive performance. We could not analyse twins who were both alive. These factors, together with sampling variability may all have contributed to the low heritability of the cognitive composite..

The sample heritability of life expectancy was .28.

The within twin-pair analysis of the life expectancy difference regressed on the intelligence difference was β=.09 (SE .06 ) *p*=.12 for the MZ twins. Among the DZ twins it was β=.29 (SE .04) p<.001. Across the whole sample the regression gave β=.22, (SE .04) p<.001. Figure S3 shows a scatterplot with a best-fit regression line that shows the slope and direction of the within pair intelligence-life expectancy relationship. Twin pairs of both zygosities show the tendency of the brighter twin to live longer, however this trend is significantly higher (*p*<.01) among the DZ twins which is consistent with a direct genetic influence mediating the two traits.

The estimates of this regression analysis were entered into the AE model to yield genetic results. The percentage of the phenotypic correlation (*r*=.35) between intelligence and life-expectancy estimated under an AE model that is explained by genes is 85.3%. The observed phenotypic correlation between intelligence and life-expectancy was 0.18

**Data considerations on the three samples.**

**Further Data Considerations**

The key limitations of the present study are small samples and (in two of the samples) late assessment of intelligence; to our knowledge there are no other genetically informative datasets that comprise intelligence measured in young adulthood as well as mortality data. Our data comprise two samples (Swedish, Danish) of men and women who entered the study in middle age at the earliest. Our samples consist of twins who may be different from singletons in ways that we do not know although twins’ survival post-infancy in Nordic populations is comparable with singletons ^2^. The intelligence composite was drawn from different tests in each sample – this matters too, because, for example, the tests administered to the Danish sample emphasize memory rather than ‘general intelligence’ – perhaps accounting for its much lower heritability since there is evidence of genetic specificity in memory ^3^. Our results may not generalize across samples where the heritability of lifespan (or intelligence) is lower because of greater environmental variance. Since we found that intelligence was negatively correlated with age at testing (in the two samples where this was known), it seems likely that our measures may index ‘cognitive decline’ in the Swedish and Danish samples to some extent. In that case, results from those samples should be interpreted as a genetic association between cognitive decline and life expectancy; however estimate of the genetic influence on the small association between intelligence and life expectancy in the NAS-NRC sample, in which intelligence was tested in young adulthood, are generally consistent with the other samples tested here. This suggests that the relationship between intelligence and life expectancy may generalize from cognitive decline to intelligence.

The NAS-NRC US military sample is all male. There may be unknown differences, relevant to this study, between men who enter the military and other men of the same age cohort; their mortality may differ from the general population since the military include some kinds of physical fitness in their entry criteria. As mentioned earlier, there is range restriction of intelligence in this sample because men at the very low end of the distribution are not admitted to the army and the mean of the sample is known to be slightly above average ^4^. Intelligence may be associated with mortality among American men who served in WWII ^5,6^. These data do not speak to that (nor to any direction of effect).

Our data do not include mortality before middle age. This reduces the variance in mortality (compared with the general population). In the Swedish and Danish samples intelligence was ascertained in later life and may represent cognitive decline. Since this is correlated with intelligence ^7^, the results remain relevant to the focal questions.

The Danish sample contains both men and women. It also only includes participants who survived to late middle age, and at first testing of cognitive ability the age range was 70 to 93 (details of ascertainment are available ^8^. This sample is similar to the Swedish sample in that cognitive decline rather than lifetime intelligence is likely being captured in the relationship with mortality.

The within-pair design makes few assumptions. A whole-sample design (that includes people born in different decades) would have to take into account the effects, known from this sample, and other studies, of a relationship between year of birth and intelligence test scores ^9^ and of sex differences in average life expectancy ^10^. These aspects of the samples (sex differences, birth year differences) make a standard bivariate ACE analysis (such as used in many behavioral genetic designs) unsuitable for our research questions. Our analysis avoids the need to control for these effects, because each comparison is made between two people of the same sex who were born on the same day.

Table S1 Descriptive Statistics of Life Expectancy and Cognitive Difference scores

| Sample | Age at death (range; mean (SD)) | Cognition (range; mean (SD)) |
| --- | --- | --- |
|  |  |  |
| NAS-NRC US military (N=377) | 0 - 25; 9.96 (5.87) | 0 - 3.62; .58 (.60) |
| Swedish (N=246) | .03 - 29.79; 8.29 (6.76) | 0 - 3.24; .77 (.67) |
| Danish LSADT (N=784) | 0 - 18.54; 6.87 (4.59) | 0 - 5.23; .87 (.73) |

NOTE: Swedish: age and sex adjusted cognition z scores

Danish: age and sex adjusted cognition z scores

US Military: unadjusted cognition z scores (no precise age, all male)

Table S2. Contingency table for Combined samples. Counts are twin pairs

|  |  |  | Lived Longer | |
| --- | --- | --- | --- | --- |
|  |  |  | No | Yes |
| MZ | Less bright | Count | 156 | 134 |
|  |  | Expected count | 146.9 | 143.1 |
|  | Brighter | Count | 155 | 169 |
|  |  | Expected count | 164.1 | 159.9 |
| DZ | Less bright | Count | 228 | 157 |
|  |  | Expected count | 190.5 | 194.5 |
|  | Brighter | Count | 152 | 231 |
|  |  | Expected count | 189.5 | 193.5 |

Table S3. Contingency table for US Military sample. Counts are twin pairs

|  |  |  | Lived Longer | |
| --- | --- | --- | --- | --- |
|  |  |  | No | Yes |
| MZ | Less bright | Count | 55 | 44 |
|  |  | Expected count | 56.1 | 42.9 |
|  | Brighter | Count | 59 | 43 |
|  |  | Expected count | 57.9 | 44.1 |
| DZ | Less bright | Count | 32 | 38 |
|  |  | Expected count | 30.8 | 39.2 |
|  | Brighter | Count | 30 | 41 |
|  |  | Expected count | 31.2 | 39.8 |

Table S4. Contingency table for Swedish sample. Counts are twin pairs

|  |  |  | Lived Longer | |
| --- | --- | --- | --- | --- |
|  |  |  | No | Yes |
| MZ | Less bright | Count | 26 | 18 |
|  |  | Expected count | 22 | 22 |
|  | Brighter | Count | 24 | 32 |
|  |  | Expected count | 28 | 28 |
| DZ | Less bright | Count | 42 | 30 |
|  |  | Expected count | 38 | 34 |
|  | Brighter | Count | 35 | 39 |
|  |  | Expected count | 39 | 35 |

Table S5. Contingency table for Danish LSADT sample. Counts are twin pairs

|  |  |  | Lived Longer | |
| --- | --- | --- | --- | --- |
|  |  |  | No | Yes |
| MZ | Less bright | Count | 78 | 62 |
|  |  | Expected count | 70.2 | 69.8 |
|  | Brighter | Count | 75 | 90 |
|  |  | Expected count | 82.8 | 82.2 |
| DZ | Less bright | Count | 154 | 89 |
|  |  | Expected count | 122.8 | 120.2 |
|  | Brighter | Count | 87 | 147 |
|  |  | Expected count | 118.2 | 1158 |

Note: These contingency tables reports the absolute value of the number of pairs in

which the brighter twin also lived longer. This is contrasted with the count expected

under the χ^2^  distribution. The χ^2^ statistics are given in the manuscript.

Table S6. Cognitive performance measures Intra Class Correlations (Confidence Intervals) between twins by zygosity

|  | MZ (*CI)* | DZ (*CI*) |
| --- | --- | --- |
| US military sample | .73 (.66 -.79) | .55 (.43 -.65) |
| Swedish sample | .82 (.75 -.87) | .33 (.20 -.44) |
| Danish LSADT sample | .43 (.34 -.52) | .31 (.22 -.39) |
| Combined samples | .60 (.55 - .65) | .34 (.28 - .40) |

Note: The intraclass correlation of the MZ twins in the Danish sample is almost half that of the other samples. This affects the heritability estimates in Table 2 main text. We assume, in this absence of other explanations, that this is owing to sample variance.

Table S7. Test of difference in slopes between zygosities. Betas, SEs and p-value of the difference

| Sample | Zygosity | beta | SE | N | *p* value of diff |
| --- | --- | --- | --- | --- | --- |
| US Military | MZ | -.145 | .08 | 223 | .013 |
|  | DZ | .137 | .08 | 154 |  |
| Swedish | MZ | .197 | .14 | 100 | .913 |
|  | DZ | .214 | .07 | 146 |  |
| Danish | MZ | .09 | .06 | 306 | .006 |
|  | DZ | .287 | .04 | 478 |  |
| Combined | MZ | .037 | .05 | 629 | .0007 |
|  | DZ | .254 | .04 | 778 |  |

Note. The random-effects meta-analysis of the MZ and DZ regression coefficients across the three samples (US, Swedish and Danish) also confirmed a significant average difference (-0.20, SD=.10, *p*=.038) between MZ and DZ pairs in the regression coefficient.

**AE Model Description and methods of within-pair regression analysis.**

Let X_ij_ be a predictor score on twin j (i =1, 2) in twin pair i, and Y_ij_ the outcome of interest. Here X and Y are intelligence and life-span, respectively. In the population, X and Y can be influenced by a number of factors, including sex, age, cohort and socio-economic status. However, for each twin pair, the effects of these factors cancel out when we consider the difference in their scores. Individual-specific effects remain and these can be genetic (A) or environmental (E). Dropping the subscript for the twin pair,

Y_1_ – Y_2_ = (A_Y1_ – A_Y2_) + (E_Y1_ – E_Y2_) and

X_1_ – X_2_ = (A_X1_ – A_X2_) + (E_X1_ – E_X2_).

The regression of Y_1_ – Y_2_ on X_1_ – X_2_ determined the covariance between the two difference scores and the variance of X_1_ – X_2_.

Var(X_1_ – X_2_) = 2(1-r_A(X)_)var(A_X_) + 2(1-r_E(X)_)var(E_X_) + 2cov(A_X1_ – A_X2_, E_X1_ – E_X2_),

with r_A(X)_ and r_E(X)_ the additive genetic and environmental correlation of the X scores in twin 1 and 2. As defined, the individual-specific environmental effects are uncorrelated (r_E(X)_ = 0) and we assume that individual-specific environmental effects are uncorrelated to the additive genetic effects. Hence,

Var(X_1_ – X_2_) = 2(1-r_A(X)_)var(A_X_) + 2var(E_X_)

For MZ twins, r_A(X)_ = 1 so Var_MZ_(X_1_ – X_2_) = 2var(E_X_), whilst for DZ twins r_A(X)_ = ½, so Var_DZ_(X_1_ – X_2_) = var(A_X_) + 2var(E_X_).

Similarly for the covariance,

cov(X_1_ – X_2_, Y_1_ – Y_2_) = 2cov_E_ for MZ twins and cov_A_ + 2cov_E_ for DZ twins, where cov_A_ and cov_E_ are the additive genetic and individual-specific covariances between X and Y in the population. These covariances can be estimated from the regression of Y_1_ – Y_2_ on X_1_ – X_2_ and the variance of X_1_ – X_2_, for MZ and DZ twins separately. Finally,

cov_E_ = ½cov_MZ_(X_1_ – X_2_, Y_1_ – Y_2_), and

cov_A_ = cov_DZ_(X_1_ – X_2_, Y_1_ – Y_2_) - cov_MZ_(X_1_ – X_2_, Y_1_ – Y_2_)

The standard errors on these estimates were calculated from the standard errors of the regression coefficients, assuming that the variance of X_1_ – X_2_ was known. Additive genetic and environmental correlations between X and Y were calculated from scaling the covariances by the additive genetic and environmental standard deviations of X and Y.


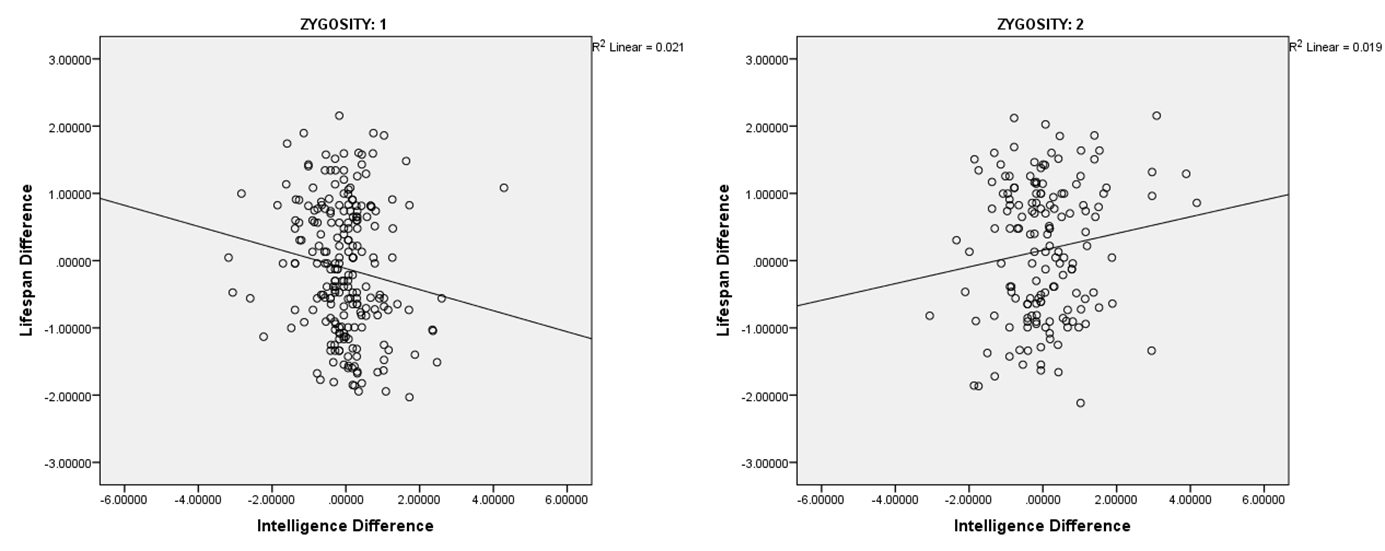


Figure S1: MZ (on left) and DZ (on right) twins: Regression of within-pair lifespan difference z-score on within-pair intelligence difference z-score (each datum = one within-pair difference score) in US military sample.


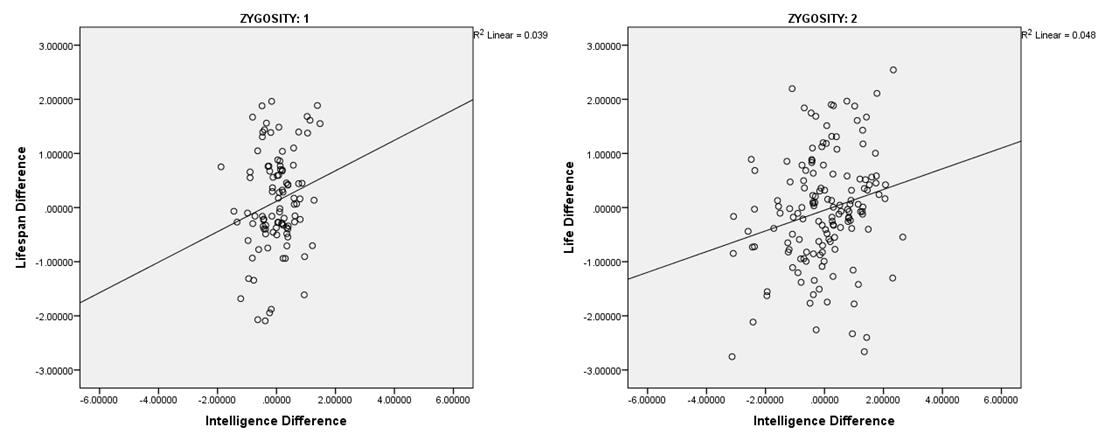


Figure S2: MZ (on left) and DZ (on right) twins: Regression of within-pair lifespan difference z-score on within-pair intelligence difference z-score (each datum = one within-pair difference score) in the Swedish SATSA sample.


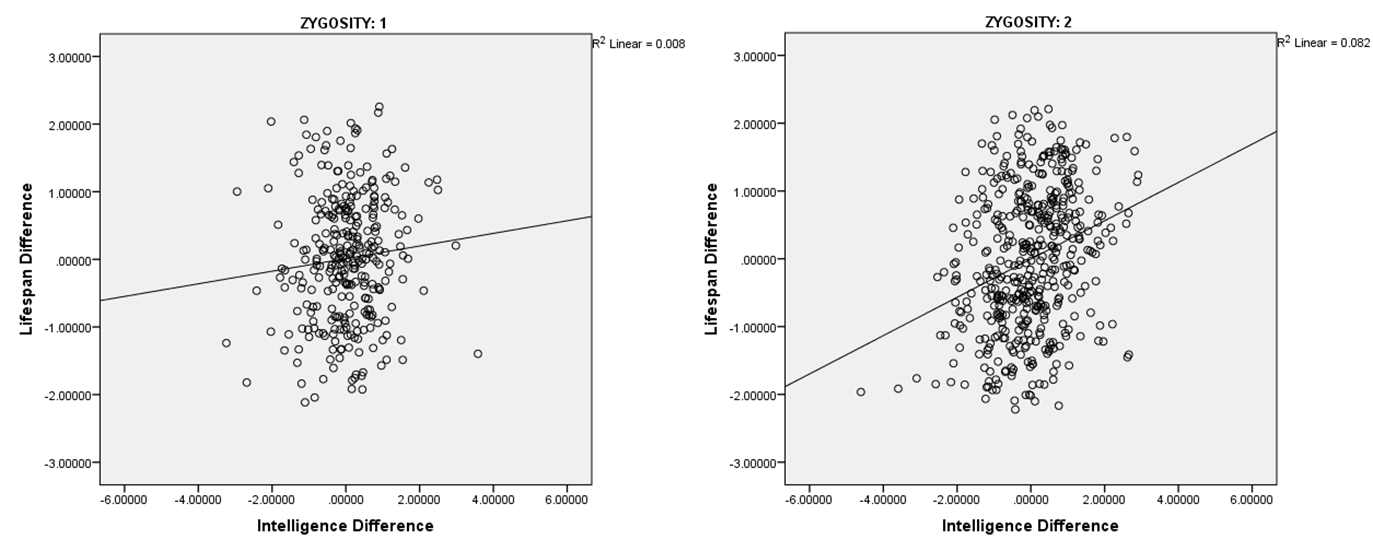


Figure S3: MZ (on left) and DZ (on right) twins: Regression of within-pair lifespan difference z-score on within-pair intelligence difference z-score (each datum = one within-pair difference score) in Danish LSADT sample.

References

1. Pedersen NL, Plomin R, Nesselroade JR, McClearn GE. A Quantitative Genetic Analysis of Cognitive Abilities During the Second Half of the Life Span. Psychol Sci 1992;3:346–53.

2. Hjelmborg JB, Iachine I, Skytthe A, et al. Genetic influence on human lifespan and longevity. Hum Genet 2006;119:312–21.

3. Kremen WS, Panizzon MS, Franz CE, et al. Genetic complexity of episodic memory: a twin approach to studies of aging. Psychol Aging 2014;29(2):404–17.

4. Plassman BL, Welsh KA, Helms M. Intelligence and education as predictors of cognitive state in late life: a 50-year follow up. Neurology 1995;45:1446–60.

5. Corley J, Crang JA, Deary IJ. Childhood IQ and in-service mortality in Scottish army personnel during World War II. Intelligence 2009;37(3):238–42.

6. Whalley LJ, Deary IJ. Longitudinal cohort study of childhood IQ and survival up to age 76. Br Med J 2001;322(7290):819–22.

7. Osler M, Avlund K, Mortensen EL. Socio-economic position early in life, cognitive development and cognitive change from young adulthood to middle age. Eur J Public Health 2013 ;23(6):974–80.

8. McGue M, Christensen K. Social activity and healthy aging: a study of aging Danish twins. Twin Rsch & Hum Genetics 2007;10 (2) 255-265.

9. Flynn JR. The mean IQ of Americans: Massive gains 1932 to 1978. Psychol Bull 1984;95:29–51.

10. Seifarth JE, McGowan CL, Milne KJ. Sex and life expectancy. Gend Med 2012;9:390–401.
